# Melatonin and Pathological Cell Interactions: Mitochondrial Glucose Processing in Cancer Cells

**DOI:** 10.3390/ijms222212494

**Published:** 2021-11-19

**Authors:** Russel J. Reiter, Ramaswamy Sharma, Sergio Rosales-Corral, Walter Manucha, Luiz Gustavo de Almeida Chuffa, Debora Aparecida Pires de Campos Zuccari

**Affiliations:** 1 Department of Cell Systems & Anatomy, Joe R. and Teresa Lozano Long School of Medicine, UT Health San Antonio, San Antonio, TX 78229, USA; 2 Centro de Investigacion Biomedica de Occidente, Instituto Mexicano del Seguro Social, Guadalajara 45150, Mexico; espiral17@gmail.com; 3 Instituto de Medicina y Biologia Experimental de Cuyo (IMBECU), Consejo Nacional de Investigaciones Cientificas y Tecnologicas (CONICET), Mendoza 5500, Argentina; wmanucha@mendoza-conicet.gob.ar; 4 Department of Structural and Functional Biology, Institute of Biosciences of Botucatu, Botucatu 18618-689, Brazil; guchuffa@yahoo.com.br; 5 Laboratorio de Investigacao Molecular do Cancer, Faculdade de Medicina de Sao Jose do Rio Preto, Sao Jose do Rio Preto 15080-000, Brazil; debora.zuccari@famerp.br

**Keywords:** melatonin, aerobic glycolysis, Warburg effect, mitochondrial metabolism, cancer, diseased cells

## Abstract

Melatonin is synthesized in the pineal gland at night. Since melatonin is produced in the mitochondria of all other cells in a non-circadian manner, the amount synthesized by the pineal gland is less than 5% of the total. Melatonin produced in mitochondria influences glucose metabolism in all cells. Many pathological cells adopt aerobic glycolysis (Warburg effect) in which pyruvate is excluded from the mitochondria and remains in the cytosol where it is metabolized to lactate. The entrance of pyruvate into the mitochondria of healthy cells allows it to be irreversibly decarboxylated by pyruvate dehydrogenase (PDH) to acetyl coenzyme A (acetyl-CoA). The exclusion of pyruvate from the mitochondria in pathological cells prevents the generation of acetyl-CoA from pyruvate. This is relevant to mitochondrial melatonin production, as acetyl-CoA is a required co-substrate/co-factor for melatonin synthesis. When PDH is inhibited during aerobic glycolysis or during intracellular hypoxia, the deficiency of acetyl-CoA likely prevents mitochondrial melatonin synthesis. When cells experiencing aerobic glycolysis or hypoxia with a diminished level of acetyl-CoA are supplemented with melatonin or receive it from another endogenous source (pineal-derived), pathological cells convert to a more normal phenotype and support the transport of pyruvate into the mitochondria, thereby re-establishing a healthier mitochondrial metabolic physiology.

## 1. Introduction

Melatonin has long been known to be an endogenously produced anti-cancer agent [1,2,3,4,5,6,7,8]. This action has been confirmed for multiple tumor types [9,10,11,12,13,14] and in both in vitro [15,16,17,18] and in vivo [19,20,21,22,23,24,25,26] studies. Similarly, many different regulatory mechanisms have been proposed to explain the ability of melatonin to restrain cancer cell proliferation, invasion and metastasis. Studies within the last decade; however, have shed new light on circadian variations in cancer cell metabolism which were not taken into account when the initially described means of cancer inhibition by melatonin were examined.

The seminal reports of Blask et al. [27], Dauchy et al. [28] and Mao and co-workers [29] provide clear evidence that, at least in some, xenografted human cancers growing in immune-compromised rats display a different metabolic phenotype during the day than at night. These studies documented that the tumors exhibited Warburg-type metabolism, a feature common to many solid tumors [28,30,31], during the day but abandoned it, in toto or in part, at night in favor of mitochondrial oxidative phosphorylation (miOXPHOS) [32,33]. Additionally, this circadian metabolic cycle is under control of the endogenous blood melatonin rhythm, which switches the cancer cells from a pathological Warburg-type metabolism during the day to a healthier metabolic phenotype at night [27,34,35]. This creates a condition where the cells primarily display a cancer phenotype during the day and are less cancerous cells at night, i.e., they are only part-time cancers [34,36].

The action of melatonin on the metabolic profile of cancer cells have far-reaching implications in terms of the mechanisms by which melatonin exhibits its oncostatic actions. Moreover, it provides essential information on the melatonin treatment strategy that could be more effectively utilized to capitalize on the anti-cancer actions of this endogenous molecule [36].

Cancer cells are by no means the only pathological cell type that adopt Warburg-type metabolism [30,37,38]. This is characteristic of many pathological cells and it is not always at the expense of abandoning miOXPHOS. The studies in question were performed using either cultured cells, which are not exposed to a circadian melatonin rhythm, or the researchers performed in vivo studies with the collection of tissue samples during the day only, when blood melatonin levels are at their nadir. Thus, the extent of cells switching from rapid ATP production during Warburg-type metabolism to miOXPHOS following exposure to melatonin on a nightly basis remains uninvestigated. This information would be important to help to define the optimal treatment time if melatonin is to be used as a medical treatment for a particular pathology [30].

Herein, the authors describe what is known about melatonin’s ability to reprogram pathological cell metabolism and the mechanism by which the circadian melatonin rhythm may interact with diseased cells to potentially alter their metabolism. We also summarize treatment paradigms of these diseases that may maximize the efficiency of melatonin in impacting these pathologies.

## 2. Melatonin: In the Right Place and at the Right Time in All Cells

For more than a decade after melatonin was discovered [39] in bovine pineal tissue, although now known to be a ubiquitously distributed molecule, it was considered unique to the vertebrate pineal gland. This image was dispelled, however, in the early 1970s, when the retina, which like the pineal gland is an ectodermal appendage of the brain, was found to produce melatonin [40,41] and as in the pineal gland, its synthesis exhibited a photoperiod-dependent rhythm [42,43]. During the same time frame, melatonin was identified in the extra-orbital Harderian gland [42], where its production also was found to be rhythmic [44]. Neither the retina nor the Harderian gland release melatonin into the systemic circulation of mammals, therefore they do not impact blood levels of the indoleamine.

The discovery of melatonin was extended to invertebrates when the compound eye of the locust (*Locusta migratoria*) was discovered to contain melatonin [45]. Melatonin was also identified in the neurosensory tissues of the gastropod mollusc (*Helix aspersa marina*) where it exhibits a diurnal rhythm [46]. Melatonin studies were taken to a more phylogenetically ancient species when melatonin was discovered in the unicellular dinoflagellate (*Gonyaulax polyedra*; now named *Lingulodinium polyedra*), where it is rhythmic, as in the vertebrate pineal gland [47,48]. An extension of these investigations to an even more primitive species was accomplished by Manchester et al. [49] when they observed immunoreactive melatonin in the photosynthetic bacterium, *Rhodospirillum rubrum*, in a photoperiod-dependent manner.

In 1995, investigations carried out by individuals associated with the same laboratory but working independently at different institutions reported the identification of melatonin in a variety of plants (both mono- and dicotyledons). Melatonin was expressed at much higher concentrations than typically measured in animals, likely due to the presence of two melatonin-producing organelles, i.e., mitochondria and chloroplasts [50,51,52,53]. Subsequently, melatonin has been identified in hundreds of plant species, in all plant organs and its synthesis, which is more complex than in animals, has been defined [54,55,56,57,58,59,60,61]. There is currently no evidence that melatonin production in plants exhibits a day-night rhythm.

Among all species, involving both animals and plants, the chemical structure of melatonin has remained constant and its actions have become progressively more widely diverse. The varied functionality of melatonin may be related to its two-to-three-billion-year evolutionary history during which it had ample time to develop complex interactions with other molecules [62]. These interchanges have allowed melatonin to express an extremely wide array of functions as exemplified in all species [61,63,64,65]. Seemingly, one of the most durable actions is its ability to suppress oxidative stress and maintain redox homeostasis in healthy cells [57,66,67,68,69,70]. In cancer cells, the actions of melatonin in terms of oxidative stress are significantly more complex, as it can function as either an antioxidant or as a pro-oxidant [71]. All aspects of oncogenesis, i.e., initiation, tumor cell survival and dissemination [72,73,74,75], are influenced by the degree of reactive oxygen species (ROS)/reactive nitrogen species (RNS) generation. Free radical-mediated oxidative stress, together with apoptosis, is often activated in tumor cells by melatonin [76].

Given that melatonin is present perhaps in all living organisms [77,78], only a small portion of which have a pineal gland (vertebrates), it is obvious that the indoleamine did not evolve as a pineal-related molecule nor is it solely derived from this organ even in vertebrates. It has been proposed, in fact, that in vertebrates, pineal melatonin represents only a small percentage (<5%) of the total melatonin generated [62]. This had already been alluded to when the total amount of melatonin in the gastrointestinal tract was calculated to be hundreds of times greater than that in the pineal gland [79].

There were several early observations that pointed to the high likelihood that, even in mammals, melatonin might be produced in greater amounts in non-pinealocytes than in pinealocytes [80]. First, melatonin was identified in many tissues, the amounts of which could not be attributed to its pineal origin. Some organs, in addition to the retina [40] and the gastrointestinal tract [79,81] that contain and were presumed to synthesize melatonin, include the cerebellum [82], thymus [83], cochlea [84], ciliary body [85], bone marrow [86], skin [87,88] and many other organs/cells [80]. Additionally, some body fluids contain concentrations that are equivalent to or higher than the maximal night time blood melatonin levels where they may or may not fluctuate in a circadian manner [89,90,91,92]. Particularly noteworthy are the exceptionally high levels of melatonin in bile [93] where it is believed to protect the cholangiocytes that line the biliary tree from toxic bile salts [94,95]. There is also evidence that melatonin in the bile reduces the incidence of gallstone formation and cholangiocarcinoma [96,97,98]. We have further reasoned that the highly elevated melatonin concentrations in the bile may in part be related to its possible enterohepatic circulation [94,98]. After its release into the duodenum, melatonin-rich bile may impact the gut microbiome [99].

Some highly inbred mouse strains are reported to be deficient in melatonin based on the inability to detect the indoleamine in the pineal [100,101]. Gomez-Corvera and colleagues [102], however, reported the presence of melatonin in immune cells obtained from two allegedly melatonin-deficient mouse strains, i.e., C57BL/6 and Swiss. The failure to detect measurable amounts of melatonin in the pineal gland as reported by Ebihara et al. [100] and Goto and colleagues [101] justifiably led to the conclusion that these mouse strains are melatonin deficient. The findings of Gomez-Corvera et al. [102], while requiring confirmation, suggest that those mouse strains may not be pan-deficient in melatonin and furthermore indicate that the genetic regulation of melatonin synthesis may differ in peripheral organs compared to the pineal gland.

In 2013, we proposed that melatonin is likely synthesized in the mitochondria (and chloroplasts) of all animal and plant cells [77]. Our rationale was based on several published findings indicating that melatonin (i) was present in almost all plant and animal species examined, (ii) exhibited multiple interactions at the mitochondrial level [88,103,104,105,106,107], (iii) was expressed at extraordinarily high concentrations in the mitochondria of hepatocytes and brain cells [108], and, (iv) acetylserotonin methyltransferase (the melatonin-forming enzyme) is localized to the mitochondrial intermembrane space in rat pinealocytes [109]. Based on the previously published studies, this speculation had credibility and was consistent with the reported existence of melatonin in a prokaryotic bacterium [49]. Bacteria are the presumed forerunners of mitochondria and chloroplasts, which evolved in early eukaryotes from α-proteobacteria and photosynthetic cyanobacteria, respectively, after they were initially phagocytized by early eukaryotes for their nutrient value [110,111]. Eventually, the engulfed proteobacteria/cyanobacteria developed a symbiotic association with the cells that engulfed them and proceeded to evolve into mitochondria and chloroplasts, respectively, which persist in all present-day eukaryotes. Considering the potential of melatonin as a free radical scavenger and its stimulation of antioxidant processes, its retention in these ROS-producing organelles was a highly fortuitous choice [105]. Whereas melatonin is a multifunctional antioxidant in healthy cells, it may also display pro-oxidant actions in pathological cells, thereby aiding in the killing of diseased cells [71,76].

## 3. Melatonin in Mitochondria: Some Assembly Required

The first experimental documentation of melatonin formation in mitochondria was reported by He et al. [112]. This group isolated and purified mitochondria from mouse oocytes, a cell in which melatonin is essential for its maturation and one in which oxidative damage must be held to a minimum to ensure a normal fetus [113,114]. Oocyte mitochondria were incubated in the presence or absence of serotonin (5-hydroxytryptamine, 5-HT), a necessary substrate for the rate-limiting enzyme, arylalkylamine N-acetyltransferase (AANAT), in melatonin synthesis. Melatonin was formed only when mitochondria were incubated with culture medium supplemented with 5-HT. Melatonin levels, measured by HPLC, increased steadily in both the mitochondria and in the culture medium for one hour during which measurements were made.

The significance of these findings is highly relevant to the putative synthesis of melatonin in mitochondria of all cells in adult mammals. All mammalian mitochondria are essentially of maternal origin, i.e., contributed by the oocyte. It seems highly unlikely that a molecule that has been preserved over billions of years of evolution and one that is of such great importance to the oocyte and other tissues [115,116] would be discarded during fetal development and organismal maturation. We surmise that melatonin production, as occurs in the mitochondria of oocytes, was passed on to all other cells during embryological development and post-natal maturation [105].

A year after the report by He and colleagues [112], a more detailed and comprehensive publication provided even stronger evidence that mitochondria produce their own melatonin. Using mouse non-synaptosomal brain mitochondria, Suofu et al. [52] identified both enzymes, arylalkylamine N-acetyltransferase (AANAT) and acetylserotonin methyltransferase (ASMT), which are involved in the conversion of 5-HT to melatonin, in the mitochondrial matrix. They also observed that melatonin synthesis in neuronal mitochondria did not exhibit a circadian rhythm as in the pineal gland, consistent with the earlier findings of Venegas et al. [108]. By knocking out AANAT, they verified the essential nature of locally produced melatonin in restraining oxidative stress in these critical organelles, at least in part. There is no evidence that melatonin produced in cellular mitochondria outside the pineal gland is released into the systemic circulation; thus, there is a releasable pool (pineal-derived) and a non-releasable pool (mitochondria of other cells) of melatonin [35].

An additional novel finding was the identification of the melatonin receptor, MT1, on the outer mitochondrial membrane [52]. The interaction of melatonin with this receptor is involved in blocking cytochrome c release from the mitochondria, which normally leads to cellular apoptosis [117,118]. Since Suofu et al. [52] found neither the AANAT nor ASMT enzyme in the cell cytosol, the conclusion was that mitochondrial matrix-generated melatonin is released and acts on its own receptor. Under in vivo conditions, given that melatonin readily enters all cells, circulating blood melatonin may also influence the MT1 receptor on the mitochondrial membrane. The findings of Suofu et al. were quickly followed by a brief report showing the presence of immunocytochemically detected MT2 receptors on mitochondria as well [119].

**Figure 1:**
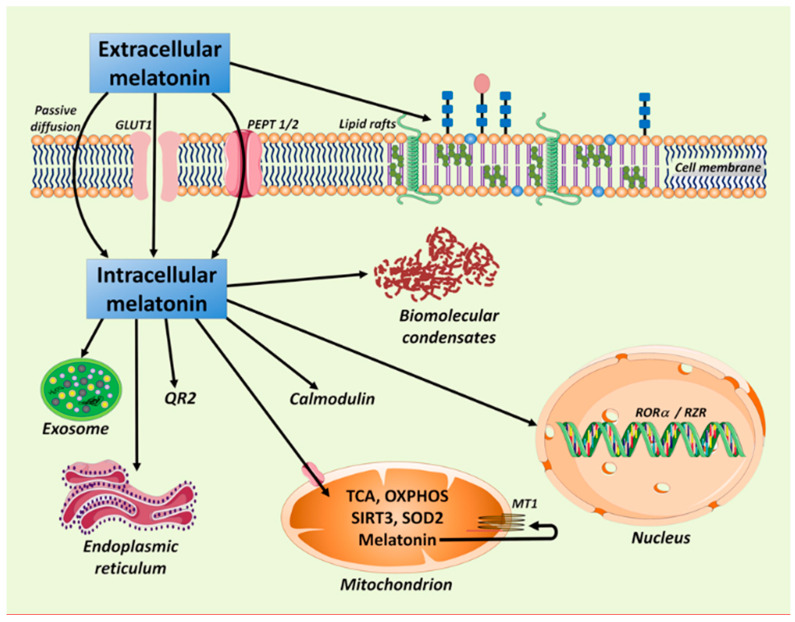
This figure summarizes the intracellular actions of melatonin. Melatonin enters cells by at least three routes, i.e., passive diffusion, GLUT1 glucose transporter (during which it reportedly competes with glucose), and via the PEPT1/2 oligopeptide transporters. Melatonin also influences lipid rafts—specialized microdomains in the cell membrane, which appear to be involved in cancer signaling. Intracellular cytosolic melatonin, either taken up from the blood or synthesized in the resident mitochondria, has multiple actions, including impacting exosome formation and cargo packaging, reducing endoplasmic reticulum (ER) stress, and binding calmodulin and quinone reductase 2 (QR2); the latter is generally referred to as MT3 melatonin receptor. In the mitochondrial matrix, melatonin influences the tricarboxylic acid cycle (TCA), mitochondrial oxidative phosphorylation (miOXPHOS), sirtuin 3 (SIRT3), superoxide dismutase 2 (SOD2), etc. Melatonin, produced in the mitochondria (as well as that imported from outside the cell) may also interact with the MT1 receptor on the mitochondrial membrane. Melatonin also enters the nucleus to bind the RORα/RZR nuclear receptors and perhaps influences the makeup of biomolecular condensates, cytosolic conglomerates of biomolecules that are involved in a variety of cell processes including cancer. Biomolecular condensates, such as lipid droplets, are not bound by a membrane.

Because of the direct receptor-independent ROS scavenging actions of melatonin as well as its interactions with intracellular receptors [56,120,121], it was speculated that melatonin readily passes through cellular and mitochondrial membranes, given its high lipophilicity. In addition to passive diffusion, melatonin reportedly enters cells via the GLUT1 glucose transporter and in doing so competes with glucose, at least in prostate cancer cells [122,123]. In addition, oligopeptide transporters, PEPT1/2, of human breast cancer cells actively transfer melatonin from outside the cell into the cytosol and from the cytosol into the mitochondria [124] (Figure 1). The active transport process allows for higher concentrations of melatonin in the mitochondria relative to the cytosol or nucleus [108,125].

## 4. Melatonin Signaling via the Cellular Membrane Receptors

As shown in Figure 1, melatonin performs several actions following its transport into the cytosol. In addition, it also interacts with cells through the membrane receptors, MT1 and MT2. These transmembrane proteins are found in many peripheral organs [126,127] and in neurons and glia of the central nervous system [128,129]. Therefore, it seems possible that every cell harbors MT1 and/or MT2 receptors. Many of the functions of melatonin in modulating oncogenic transformation are mediated by signaling events that follow the binding of melatonin to its membrane receptors [2,7,130,131].

**Figure 2:**
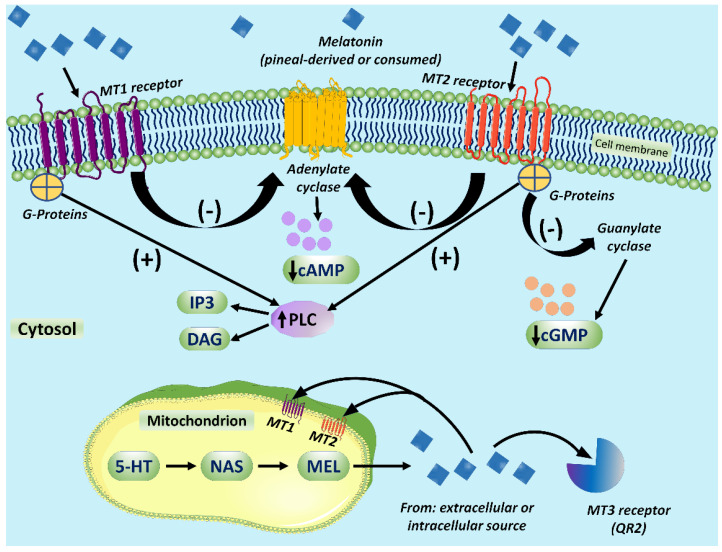
This figure illustrates the intracellular signaling processes of MT1 and MT2 receptors. Most, perhaps all, cells possess the major melatonin membrane receptor subtypes, MT1, MT2 and MT3. These receptors are members of the G-protein coupled receptor (GPCR) family and have been well characterized pharmacologically and cloned. The receptors are differentially distributed depending on the specific cell type and, in general, MT1 seems to be more common than MT2. Also shown are the MT1 and MT2 receptors on the mitochondrial membrane and the cytosolic MT3 receptor, quinone reductase 2 (QR2). Since the identification of the receptors on the mitochondria is a recent finding, little is known about their signaling pathways. 5-HT = serotonin; c-GMP = cyclic guanosine monophosphate; DAG = diacylglycerol; IP3 = inositol triphosphate; MEL = melatonin; NAS = N-acetylserotonin; PLC = phospholipase C.

The MT1 and MT2 receptors are members of the G-protein coupled receptor (GPCR) family [131,132,133]; they have the requisite seven transmembrane domains and are linked to a variety of signaling processes [134,135]. Abundant studies have confirmed that melatonin binding to these receptors modulates cAMP production, phosphorylation, morphological adaptation, and intracellular calcium mobilization (Figure 2) [126,136]. When melatonin binds to MT1 receptor, which generally appears to have a wider distribution than the MT2 receptor [127], it suppresses forskolin-stimulated cAMP production, reduces protein kinase A (PKA) activity and depresses phosphorylation of the cAMP responsive element binding protein (CREB) [137]. These changes lead to the triggering of ERK1/2, enzymes committed to cytoskeletal filament remodeling (Figure 2) [138].

Binding of melatonin to the MT2 receptor also leads to a drop in cAMP and a stimulation of protein kinase C and phospholipase C [139]. The upregulation of G-proteins also impacts membrane permeability enhancing the opening of ion channels [140]. Concurrently, cGMP is elevated, which stimulates opening of cyclic nucleotide-gated channels allowing calcium influx. The MT2 receptors are of special interest to chronobiologists, as their binding to melatonin results in multiple circadian rhythm and phase shifting actions [141,142]. Melatonin also impacts transcriptional events and gene expression because of its ability to influence CREB and ERK signaling [143].

MT1 and MT2 can form homo- or heterodimers that signal intracellular processes via the canonical G-proteins, i.e., α_i_, α_i2_, α_i3_, β and δ [144]. Many of the signaling events underlying the membrane-mediated actions of melatonin are summarized in Figure 2. The MT1 and MT2 receptors are involved in a multitude of physiological and pathophysiological processes, including glucose metabolism and cancer progression [35,65,130,145,146].

There are several perturbations that significantly reduce circulating melatonin levels, which, as a result, negate the important signaling processes of the melatonin receptors. Advanced age is often highly detrimental to the production of melatonin in the pineal gland [147,148] and possibly also at the mitochondrial level in all cells [26,149], especially in the frail elderly. Because of the requirement for darkness at night to ensure pineal, but not mitochondrial, melatonin production, light pollution is a major factor in the suppression of blood melatonin levels, contributing to circadian disturbances and carcinogenesis [150,151,152]. Interruption of the neural connections between the master circadian oscillator, the suprachiasmatic nucleus (SCN), and the pineal gland inhibits melatonin synthesis; this has been demonstrated in quadriplegics [153] and after cervical spinal cord lesions that involve destruction of the cephalic sympathetic neurons [154,155]. Finally, a number of diseases are associated with diminished blood melatonin levels either due to its reduced synthesis or rapid uptake and utilization [29,156]. In each of these situations, the receptor signaling is lost or reduced, which contributes to aberrant cell metabolism and pathophysiology [65,119,157]. Since the MT1/MT2 receptors seem to be present in tissues of aged animals, melatonin supplementation may still have efficacy in regulating cellular metabolism and functions in the elderly [158].

## 5. Melatonin in Mitochondria: Relation to Oxidative Stress and Glucose Metabolism

**Figure 3:**
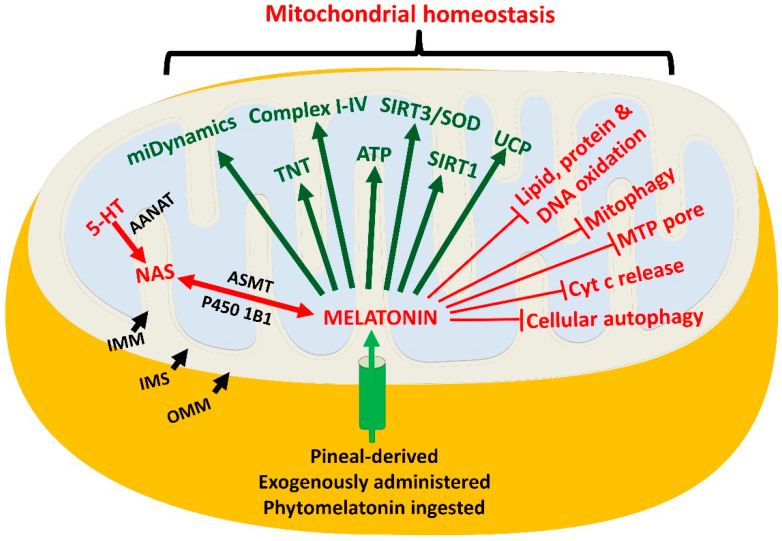
Representative actions of melatonin that involve the mitochondria. Melatonin, derived from the pineal gland, after supplemental ingestion or consumed in the diet is taken up by cells and transported into the mitochondria via the oligopeptide transporters, PEPT1/2. All cells are believed to synthesize melatonin in their mitochondria via the conventional pathway as described in the pineal gland. In mitochondria, melatonin can be reverse-metabolized to its precursor, N-acetylserotonin (NAS); this involves the extrahepatic monooxygenase enzyme, P450 1B1. Thus, the changes induced by melatonin may also involve NAS production. The most recently discovered actions of melatonin that involve the mitochondria are its effects on tunneling nanotubes (TNT) which allow for the transfer of mitochondria between cells. 5-HT = serotonin; AANAT = arylalkyl-N-acetyltransferase; ASMT = acetyl serotonin methyltransferase; Cyt c = cytochrome c; IMM = inner mitochondrial membrane; IMS = Intermembrane space; miDynamics = mitochondrial dynamics; MTP = Mitochondrial permeability transition pore; OMM = outer mitochondrial membrane; UCP = uncoupling protein; SOD = superoxide dismutase.

**Figure 4:**
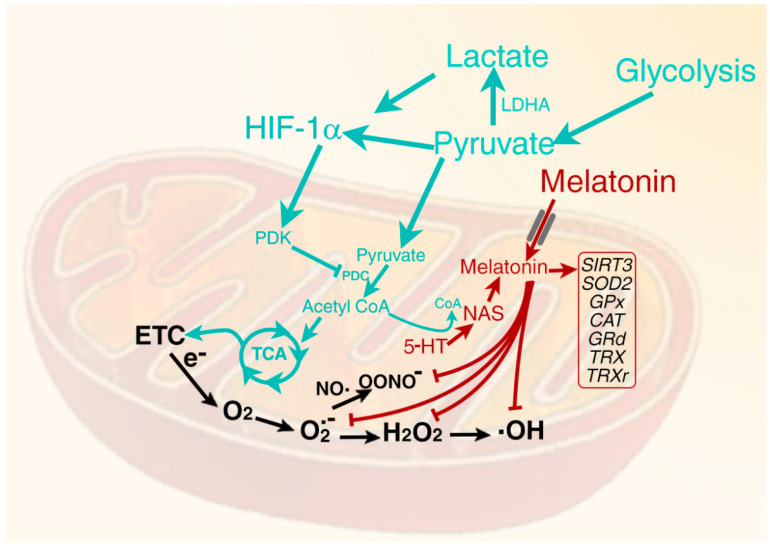
Melatonin is synthesized in mitochondria and can be taken up from the systemic circulation by these organelles. In healthy cells, mitochondrial melatonin is an effective direct scavenger of ROS and RNS (shown by red lines), which are generated normally but also in excess when the electron transport chain (ETC) is faulty. Additionally, melatonin indirectly aids in maintaining redox homeostasis by upregulating the deacetylase, SIRT3, and a series of antioxidant enzymes (boxed). This figure also illustrates the necessity of acetyl-CoA for mitochondrial melatonin production; melatonin synthesis ceases in the absence of this co-substrate. The absence of acetyl-CoA is a consequence of the inhibition of the pyruvate-to-acetyl-CoA transformation due to the suppression of pyruvate dehydrogenase complex (PDC; PDH is one component of this complex), which is under tight control, and inhibited by pyruvate dehydrogenase kinase (PDK). The upregulation of PDK is often a result of the stabilization of cytosolic hypoxia-inducible factor-1 α (HIF-1α), an oxygen-sensing transcription factor. In normoxic healthy cells, HIF-1α is ubiquitinated and undergoes proteasomal degradation. During hypoxia, HIF-1α is stabilized, leading to the upregulation of PDK and the inhibition of PDH, resulting in mitochondrial melatonin synthesis inhibition. Warburg metabolism (aerobic glycolysis), however, occurs under normoxic conditions; in this case, the excess of pyruvate and lactate in the cytosol presumably destabilizes HIF-1α such that PDH is disinhibited. 5-HT = serotonin; CAT = catalase; CoA = coenzyme A; e^−^ = electron; GPx = glutathione peroxidase; GRd = glutathione reductase; H_2_O_2_ = hydrogen peroxide; LDHA = Lactate dehydrogenase-A; NAS = N-acetylserotonin; NO· = nitric oxide; ·OH = hydroxyl radical; ONOO^−^ = peroxynitrite anion; PDH = pyruvate dehydrogenase; SOD2 = superoxide dismutase 2; TCA = tricarboxylic acid cycle; TRX = thioredoxin; TRXr = thioredoxin reductase.

Mitochondria are multifunctional organelles involved in almost every cellular activity (Figure 3), i.e., autophagy, apoptosis, glucose metabolism, energy production, etc. [159,160,161]. The latter function is related to their role in ATP production. During the process of ATP generation, the transfer of electrons between successive proteins of the electron transport chain (ETC) is not flawless. Some electrons reduce adjacent ground state oxygen (O_2_) [162,163] to the superoxide anion radical (O_2_^.−^). O_2_^.−^ is itself damaging to neighboring healthy molecules but is rapidly metabolized to the hydroxyl radical (^.−^OH) and the peroxynitrite anion (ONOO^−^), which results in even greater molecular destruction. Normally functioning healthy cells are equipped with a variety of antioxidants, which keep molecular damage to a minimum but still allow ROS to function in essential signaling pathways [164,165]. In normal cells, the redox homeostasis is maintained by a large number of antioxidant enzymes, which convert toxic species to less harmful derivatives, thereby reducing oxidative stress (Figure 4). The recent discovery of melatonin in mitochondria [52,112] of healthy cells and its synthesis in these organelles contributes to limiting ROS destruction as melatonin directly scavenges ROS [71,109,166,167,168] and also stimulates antioxidant enzyme expression. Many studies have confirmed the ability of melatonin to reduce damage to key mitochondrial constituents including proteins of the ETC and the mitochondrial genome [65,168,169], which normally appears under conditions of high oxidative stress. Melatonin’s ability to stimulate mitochondrial superoxide dismutase 2 (SOD2) follows its deacetylation signaled by upregulation of the major mitochondrial deacetylase, SIRT3 [168,170,171].

In addition to or as a result of exposure to excessive ROS/RNS, many pathological cells adopt an alternative method to more rapidly generate ATP to support their metabolism; this involves upregulating glycolysis and rewiring pyruvate metabolism. As an end product of glycolysis, pyruvate is transported into the mitochondria in normally functioning cells, where it is irreversibly decarboxylated to acetyl coenzyme A (acetyl-CoA) [172]. This important product enters the tricarboxylic (TCA)/citric acid cycle (CAC) and eventually improves the efficiency of the ETC and miOXPHOS [173]. Additionally, as noted above, acetyl-CoA is a prerequisite for AANAT to metabolize 5-HT to NAS when it donates its acetyl group for the formation of NAS, which is subsequently converted to melatonin (Figure 3 and Figure 4). Downregulation of the gatekeeper enzyme, PDH by pyruvate dehydrogenase kinase (PDK) [174] requires pyruvate to be metabolized by an alternate cytosolic pathway, thereby impeding mitochondrial production of melatonin and modulation of its downstream processes (Figure 3). The replacement pathway for pyruvate is its enzymatic conversion to lactate in the cytosol. This change is associated with enhanced glucose uptake by cells and accelerated glycolysis, which also rapidly, but in relatively low yield, generates ATP [175]. Due to the large amounts of lactate produced under these conditions, much of it is discharged from the cell via the monocarboxylate transporter leading to the acidification of the tumor microenvironment. In the case of cancer cells, an acidic microenvironment aids cellular aggressiveness, invasion and metastasis [176].

The absence of melatonin synthesis in the mitochondria significantly alters their physiology due to its multitude of functions in these organelles (Figure 3) [105,146]. One of the major roles of melatonin in healthy cells is to maintain mitochondrial redox hemostasis, in part, by scavenging ROS and RNS when the cells are challenged with an oxidant [103,104]. The presence of melatonin would be even more important under pathological conditions [177], especially in cells manifesting Warburg-type metabolism. Excess O_2_*^.−^* would be formed in these cells due to the less efficient ETC leaking additional electrons. O_2_*^.−^* is the first in a chain of partially reduced oxygen and nitrogen-based derivatives that inflict damage at the mitochondrial level (Figure 4).

Warburg metabolism (aerobic glycolysis) is common to many pathological cell types. This process involves the rapid uptake of large amounts of glucose and accelerated glycolysis, with the end product, pyruvate, metabolized to lactate rather than entering mitochondria for conversion by PDH to acetyl-CoA. Among the many features that make Warburg metabolism unique is that it occurs in the presence of optimal intracellular oxygen concentrations.

This metabolic phenotype seems to be rather labile and can be abandoned quickly, presumably in the favor of conventional miOXPHOS, although Warburg-type metabolism and OXPHOS can also co-exist. The rapidity with which it may be reversed is highlighted by Blask and coworkers [27]. They observed in vivo that breast cancer cells exhibited Warburg-type metabolism during the day but not at night when lactate production was markedly reduced. This switch was governed by the nocturnal rise in serum melatonin, as the metabolic change did not occur when the night time increase was prevented by exposing animals to light at night. Since Warburg-type metabolism is typically associated with diseased cells, the findings of Blask et al. [27] and others [28,29] imply that these cancer cells manifest a pathological phenotype during the day but a healthier phenotype at night, as long as melatonin from the pineal gland is available [30,34].

The dependence of this day-to-night shift on the availability of circulating melatonin is confounded by the observation that mitochondria of possibly all cells produce melatonin in a non-circadian manner [52,108]. To date, only the mitochondria of normal/healthy cells have been investigated for their melatonin-synthesizing ability [52,112]. These organelles have the enzymes required to convert 5-HT to NAS and to transform NAS into melatonin. Any metabolic process that deprives mitochondria of pyruvate may also eliminate intramitochondrial melatonin production since no acetyl-CoA would be available as a co-substrate for 5-HT in the melatonin synthetic pathway [178,179,180]. Such a scenario occurs in a variety of pathological conditions in addition to cancer [181].

Many solid tumors adopt Warburg-type metabolism. Under these circumstances, pyruvate is precluded from entering the mitochondria, as PDH, which converts it into acetyl-CoA, is strongly downregulated. PDH is a complex of mutually dependent enzymes, one of which is pyruvate dehydrogenase E1α. PDH E1 α is inhibited by another mitochondrial enzyme, PDK [182]. Because of this series of events, melatonin is presumably not synthesized in the mitochondria of cells utilizing Warburg-type metabolism in the absence of acetyl-CoA. As a result, pyruvate is retained in the cytosol where it is converted to lactate by lactate dehydrogenase A [183].

There are a host of diseased/pathological conditions in which mitochondria-derived free radicals are implicated in contributing to the malfunctioning of cells. Examples of oxidative damage-related pathologies are amply evidenced in the literature. Included in this list is ischemia-reperfusion injury resulting from transient vascular occlusion [184,185], hypertension [186], cancer initiation and progression [46,187], neurodegeneration [188], sepsis [189,190], radiation injury [191], metabolic syndrome [192,193] and many others. Without exception, at least experimentally, melatonin mitigates the severity of mitochondrial dysfunction in each of these conditions [107,194,195,196,197]. Whether the mitochondria of these damaged cells are capable of producing their own melatonin reserves has yet to be examined, but this is rather unlikely given the above data.

## 6. Role of Hypoxia Inducible Factor in Determining the Metabolic Phenotype

The upregulation of the hypoxia inducible factor-1α (HIF-1α) is frequently responsible for a reduction in the intramitochondrial conversion of pyruvate to acetyl-CoA, which would compromise melatonin synthesis in these organelles. HIF-1α is a critical oxygen-sensing transcription factor. It responds to low oxygen tension by adjusting the physiology of the cells using multiple mechanisms. These include promoting glycolysis, stimulating the pentose phosphate pathway, supporting angiogenesis, and, inducing the release of lactate from the cell, thereby making the extracellular microenvironment more acidic. In the case of cancer cells, these metabolic adjustments enhance tumor growth, invasion and metastasis.

Since the ingrowth of blood vessels into the tumor lags behind the rapid proliferation of cancerous cells, some tumor cells become hypoxic and invariably suffer from oxygen deficiency. This stabilizes HIF-1α, which then rewires cellular metabolism to a phenotype that provides advantages to pathological cells in terms of tumor growth and metabolism. Examples of such advantages include rapid ATP availability and metabolites required to fuel the accelerated development.

The change in the rate of glycolysis and the shunting of pyruvate to lactate is often a result of intracellular hypoxia. Low oxygen tension stabilizes the transcription factor, HIF-1α, which, in turn, upregulates PDK, causing inhibition of PDH and consequent failure of mitochondrial acetyl-CoA formation [198]. This alternate metabolism can also occur when cells are normoxic, a metabolic phenotype discovered over a century ago and named aerobic glycolysis (the Warburg effect) [199]. Under these conditions of normal oxygen tension, pyruvate is not converted to acetyl-CoA in the mitochondria [200,201]. Finally, melatonin has reportedly been shown to be a direct inhibitor of HIF-1α [202,203,204,205]. Thus, during aerobic glycolysis, mitochondrial melatonin synthesis is presumably depressed, as no acetyl-CoA is available to support its synthesis. A lack of melatonin prevents inhibition of HIF-1α such that PDH suppression persistently dampens its production, thereby allowing for the continuation of Warburg metabolism. Clearly, a number of processes may synergize to sustain aerobic glycolysis.

Stabilization of HIF-1α during aerobic glycolysis in cancer cells may also be, in part, related to the loss of mitochondrial melatonin synthesis owing to its antioxidant activity, while also reducing cytokine production and/or release [206,207,208,209]. When melatonin neutralizes these biomolecules, HIF-1α is destabilized and normal mitochondrial metabolism reinstituted [34,146]. This possibility is supported by the findings of Blask et al. [27], Dauchy and coworkers [1], and Mao et al. [29] who found that the presence of melatonin switched metabolism in cancer cells away from aerobic glycolysis.

Under normoxic conditions, HIF-1α is rapidly degraded by the proteasome after its ubiquitination [210,211]. However, normoxia is not always associated with the early destruction of HIF-1α, thus allowing it to function as it does under low oxygen tension. Therefore, pyruvate to lactate conversion proceeds abundantly, resulting in a failure to synthesize mitochondrial acetyl-CoA, characteristic of aerobic glycolysis. Agents that prolong the stability of HIF-1α include RNS, especially NO^.^, ROS, growth factors and a number of cytokines [212]. Additionally, the end products of glycolysis, pyruvate and lactate, also function to directly stabilize HIF-1α ensuring the persistence of Warburg metabolism even under conditions of normoxia (Figure 4) [200,201,204].

## 7. Concluding Remarks and Perspectives

There is an intensive search for therapeutic agents that will promote normal miOXPHOS in cells undergoing Warburg-type metabolism. The best known of these molecules is dichloroacetate (DCA), which is also used as an anticancer treatment [213,214]. DCA redirects pyruvate into the mitochondria by destabilizing HIF-1α, which subsequently disinhibits PDH in the mitochondrial matrix, allowing for the conversion of pyruvate to acetyl-CoA. This enhances downstream events, such as the TCA cycle and the functions of the ETC, and likely contributes to the oncostatic effects of DCA [213]. Melatonin is an endogenously synthesized molecule with similar actions to those of DCA; it also reverses Warburg-type metabolism and functions as an anticancer agent. This action of melatonin also involves modulation of the HIF-1α/PDK/PDH axis. Unlike DCA [214], melatonin lacks toxicity and has a very high safety profile at any dose [215].

Regarding the treatment of melatonin-sensitive tumors, the circadian biology of cancer cells has implications at the clinical level [36]. The field of chronopharmacology, i.e., treating cells at the most efficacious time in the constantly changing 24 h metabolism of pathological cells, has a long history [216,217,218,219]. When melatonin is used as a treatment, oncologists could capitalize on the apparent fluctuation in metabolism specific to a given cancer type. In the current review, we mention that at least one breast cancer subtype [87], and likely other tumor types, alternate between Warburg-type metabolism and miOXPHOS during each 24 h period. This cycle requires a night time rise in circulating melatonin levels. Since Warburg metabolism appears to occur in pathological cells, we suggest that when melatonin is used as an oncostatic agent, it should be prescribed when the tumors are undergoing aerobic glycolysis, i.e., during the day. This would likely be the time at which cancers would exhibit the greatest response to oncostatic agents such as melatonin. Melatonin could also be given concurrently with other chemotherapies, as it does not interfere with the anti-cancer effects of these agents but it does reduce their collateral toxicity [220,221,222]. Moreover, treating human tumors with melatonin reverses their resistance to chemotherapeutic agents, such as tamoxifen [223]. In individuals who lack a melatonin rhythm, e.g., in the frail elderly, melatonin treatment could be extended throughout the 24 h period. Due to the advances in melatonin delivery systems, treatment with melatonin for prolonged periods is feasible [224].

The identified actions of melatonin at the mitochondrial level have primarily accumulated within the last two decades, with several of them being elucidated only within the last several years. The burgeoning number of investigations into mitochondrial functions of melatonin is likely due to its unusually high concentrations in this organelle [108] as well as the speculation that mitochondria of all cells produce melatonin [77]. These findings have aided in defining the unusually wide-ranging actions of this ubiquitously distributed molecule. Despite many studies directed at elucidating the antioxidant [106,168,225,226,227], anticancer [25,76,228,229,230], and chronobiological (including sleep) actions [142,219,231,232,233] of melatonin, there are many other functions of melatonin that warrant further investigation, particularly those related to disease prevention and biological aging.

Mitochondrial malfunction is common in innumerable disturbed cellular activities [106,168,234,235,236] and in many diseases [237,238,239,240]. The optimal functioning of mitochondria stems, at least in part, from the local availability of melatonin (Figure 3). It is conceivable that mitochondrial function is maintained by melatonin produced and secreted from the pineal gland, which is then actively taken up by the mitochondria of all cells. However, as noted before, a major drawback of this hypothesis is the rather small quantities of melatonin released from the pineal gland, which would be inadequate to cater to the trillions of mitochondria in every organism. Moreover, melatonin is exclusively released from the pineal gland at night such that, for every 24 h period, mitochondria would only have access to melatonin about half of the time. This would seem to be incompatible with the function of these organelles, as they are involved in a wide range of essential diurnal activities. The non-reliance of mitochondria on melatonin released from the pineal gland is also consistent with the high concentrations of mitochondria in plant cells [67,241,242], which are probably not dependent on melatonin delivered from another organ.

The necessity for the mitochondrial synthesis of melatonin likely also comes into play with respect to the specific activity pattern a species displays. Vertebrates are classified as nocturnal, diurnal or as having a crepuscular activity pattern. If well-functioning mitochondrial physiology indeed relies on melatonin supplied by the pineal gland, these organelles may function suboptimally during the daily interval when ATP synthesis is at the peak in diurnally active species. To ensure highly functioning mitochondria, every vertebrate species (diurnal, nocturnal or crepuscular) may have opted for the continual mitochondrial production of this critical constituent throughout each 24 h period in normal cells [52,108]. On the contrary, reduced synthesis of mitochondrial melatonin may lead to the development of pathological conditions. The specific overt activity patterns of species summarized above are certainly under the influence of the circadian cerebrospinal fluid (CSF) and blood melatonin rhythms which regulate clock genes in the SCN [210,243,244], as well as peripheral oscillators mediating crucial chronological functions in all cells [245,246,247,248], respectively. The hub of melatonin interactions with glucose metabolism in cells obviously involves the regulation of mitochondrial acetyl-CoA synthesis. Reconciling the interactions of circulating melatonin with melatonin produced in the mitochondria requires further detailed investigation. The data accumulated to date show that melatonin from both sources, pineal-derived and mitochondria, likely play critical roles in regulating mitochondrial physiology. This notion is consistent with the numerous effects of melatonin, not only on glucose processing, but also on other diverse cellular actions. Finally, identifying which of the many actions of melatonin are mediated by their known receptors and which are receptor-independent obviously requires more complete detailed examination.

## Data Availability

No data included.
